# Can bending radiographs be useful for Lenke classification system of idiopathic scoliosis?

**DOI:** 10.1186/1748-7161-8-S1-O28

**Published:** 2013-06-03

**Authors:** M Tyrakowski, T Kotwicki, A Koch, M Drwiega, K Lisiecka, D Lawniczak, S Pietrzak, J Czubak

**Affiliations:** 1 Department of Orthopaedics, Pediatric Orthopaedics and Traumatology of Postgraduate Medical Education Centre in Warsaw, Poland; 2 Spine Disorders Unit, Department of Pediatric Orthopedics and Traumatology, University of Medical Sciences, Poznan, Poland

## Background

Lenke classification of idiopathic scoliosis (IS) is based on standing ap and lateral radiographs, combined with assessment of scoliosis correction on bending X-rays.

## Aim

The aim of this study was to verify the usefulness of bending films for Lenke classification of IS.

## Methods

The radiographs of 30 consecutive patients operated on because of IS were examined. Seven independent researchers assessed the X-rays, in 3 stages at one week intervals.

Stage 1: Lenke type was determined on AP and lateral long film standing X-rays.

Stage 2: Lenke type was established by use of AP and lateral standing X-rays, completed with supine traction films.

Stage 3: Lenke type was indicated using AP and lateral standing, supine traction, and lateral bending films.

The order of the radiographs, in each stage, was different and random. The results were determined by calculating the inter-observer and intra-observer agreement, and were quantified using two-rater and multi-rater kappa statistics.

## Results

The inter-rater agreement in the first, and second, stage was moderate (multi-rater kappa coefficient: 0.41 in the first stage and 0.43 in the second stage; percentage agreement: 0.56 and 0.55 respectively). The inter-rater agreement in the third stage was less (multi-rater kappa coefficient: 0.34 and percentage agreement: 0.47). The intra-observer agreement was the highest between the first and second stage for each of the researchers (mean kappa coefficient: 0.69; mean percentage agreement: 0.76).

## Conclusions

The use of lateral bending X-rays in classifying the IS, according to the criteria of Lenke, reduced the intra-rater and the inter-rater agreement. Supine traction radiographs of the spine improved the agreement. This may suggest that the supine traction X-rays may help in classifying the IS.
